# Correction: Identification of an optimal exogenous gene insertion site (P–M) and establishment of a reverse genetics system for aMPV/A

**DOI:** 10.1371/journal.pone.0358322

**Published:** 2026-09-11

**Authors:** Yu Guo, Ting Xue, Bingqing Lu, Jiqing Zheng, Yirong Wang, Xueqiao Xie, Huiting Wang, Furui Cao, Yijun Zhang, Jianhua Wang

In Fig 4, the labels on the right side of A, “raMPV/B-PM-EGFP P5, 10, 15, 20”, have been changed to “raMPV/A-PM-EGFP P5, 10, 15, 20” and the label “raMPV-C-HA” in B has been changed to “raMPV/A-EGFP”. Please see the correct Fig 4 here.

**Fig 4:**
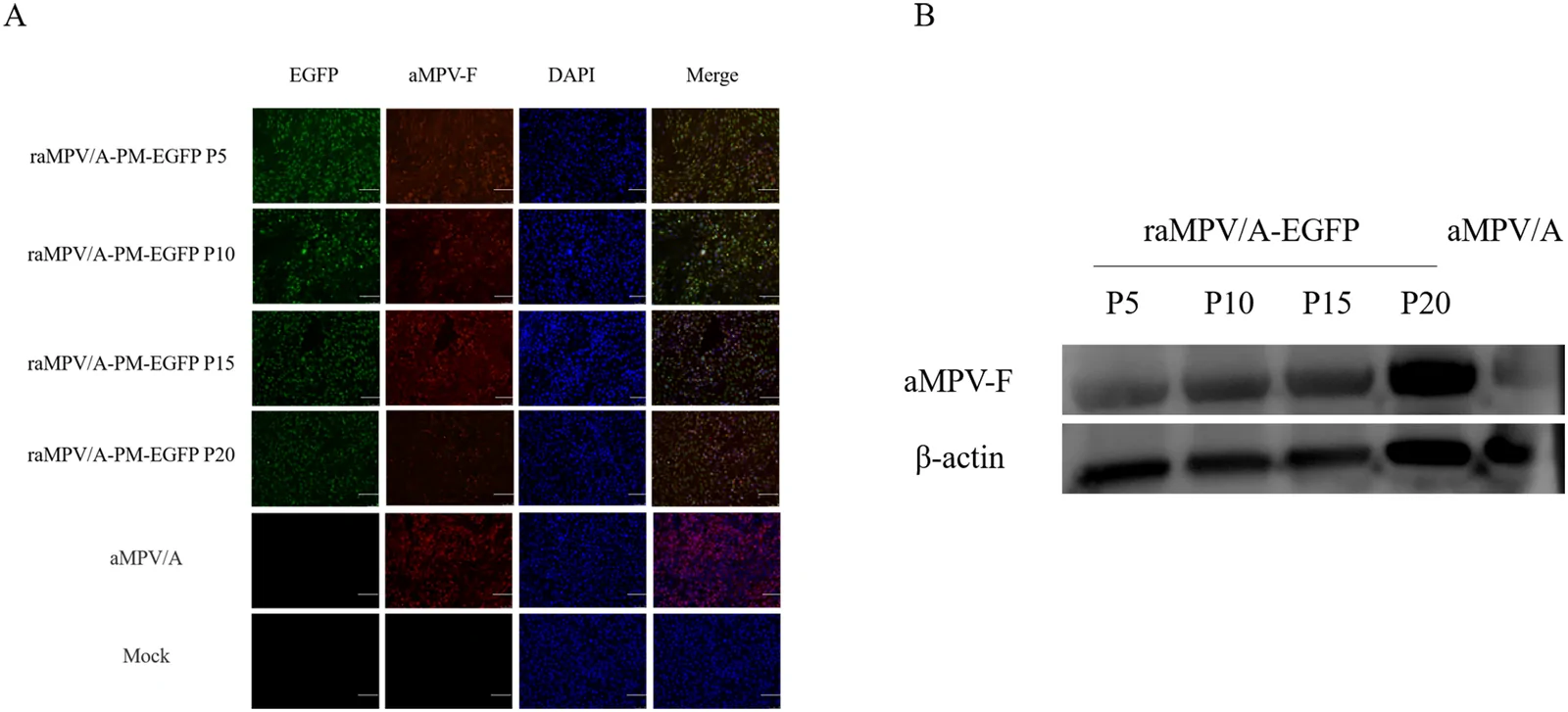
Expression of EGFP in different generations of aMPV/A-PM-EGFP recombinant viruses. Genetic stability analysis of raMPV/A-PM-EGFP (the optimal recombinant strain) after serial passage (P5, P10, P15, P20; passage 5, 10, 15, 20) in Vero cells, with parental aMPV/C as the negative control and chicken β-actin as the internal reference protein. A Indirect immunofluorescence assay (IFA) for the detection of aMPV-F protein expression in raMPV/A-PM-EGFP at different passages. Scale bar = 100 μm. B Western blotting (WB) analysis of aMPV-F protein and EGFP expression in serially passaged raMPV/A-PM-EGFP. Primary antibodies included a monoclonal antibody against aMPV-F, a specific antibody against EGFP, and a monoclonal antibody against chicken β-actin. Lanes are as follows: 1 = parental aMPV/C (negative control); 2 = raMPV/A-PM-EGFP P5; 3 = raMPV/A-PM-EGFP P10; 4 = raMPV/A-PM-EGFP P15; 5 = raMPV/A-PM-EGFP P20.
