# Supplementary figures and images for: Identification of an optimal exogenous gene insertion site (P–M) and establishment of a reverse genetics system for aMPV/A
Source: PLoS One. 2026 Apr 20;21(4):e0347597. doi: 10.1371/journal.pone.0347597 (PMC13094954; doi:10.1371/journal.pone.0347597)

|      |  |                   |     |     |               |
|------|--|-------------------|-----|-----|---------------|
|      |  | <u>raMPV/C-HA</u> |     |     | <u>aMPV/C</u> |
| Make |  |                   |     |     |               |
|      |  | P5                | P10 | P15 | P20           |

aMPV F 70 KDa

β-actin     42 KDa

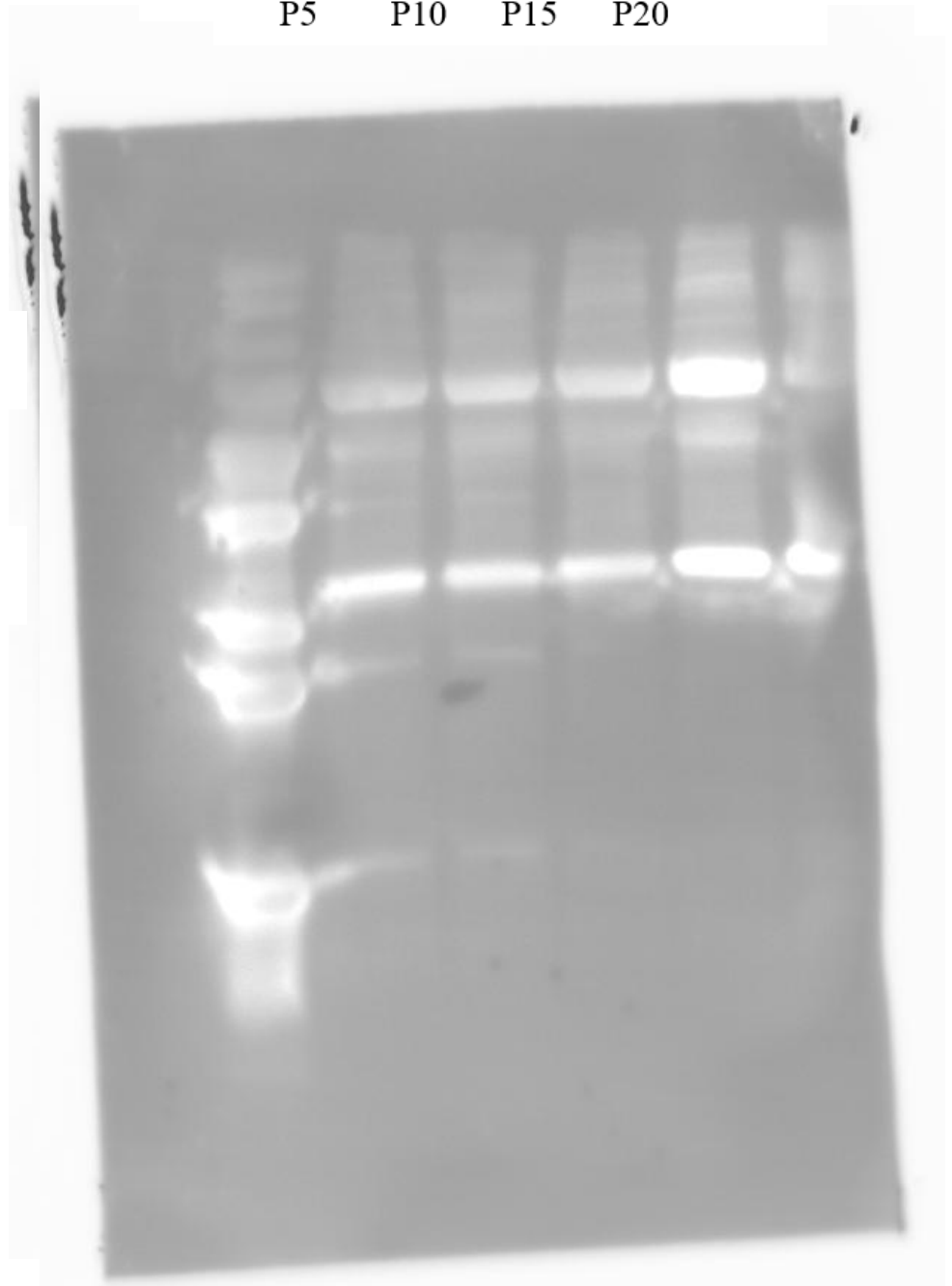

Supplement: S1 File — The original WB images used in the research. (PDF) [file pone.0347597.s002.pdf]
