# Supplementary material for: Identification of an optimal exogenous gene insertion site (P–M) and establishment of a reverse genetics system for aMPV/A
Source: PLoS One. 2026 Apr 20;21(4):e0347597. doi: 10.1371/journal.pone.0347597 (PMC13094954; doi:10.1371/journal.pone.0347597)
Supplement: Attachment — Submitted filename: Response to Reviewers.docx [file pone.0347597.s005.docx]

**Response to Reviewers**

**Response to academic editor：**

1. **Please ensure that your manuscript meets PLOS ONE's style requirements, including those for file naming.**

Reply：The manuscript has been revised according to the format of PLOS ONE. All the modifications have been marked as the highlighted yellow parts in the text.

2. **Please note that PLOS One has specific guidelines on code sharing for submissions in which author-generated code underpins the findings in the manuscript.**

Reply：There is no information about code in my research.

3. **We note that the grant information you provided in the ‘Funding Information’ and ‘****Financial Disclosure’ sections do not match.**

Reply：I have revised the Financial Disclosure based on the content of Funding Information.

4. **Please state what role the funders took in the study. If the funders had no role, please state: "The funders had no role in study design, data collection and analysis, decision to publish, or preparation of the manuscript."**

Reply：Funder statement：The funders had no role in study design, data collection and analysis, decision to publish, or preparation of the manuscript.

5. **Please note that funding information should not appear in any section or other areas of your manuscript. We will only publish funding information present in the Funding Statement section of the online submission form. Please remove any funding-related text from the manuscript.**

Reply：The funding information has been deleted.

6. **Please confirm at this time whether or not your submission contains all raw data required to replicate the results of your study.**

Reply：All the original data have been supplemented and uploaded to the supplementary file.

7. **In your cover letter, please notewhether your blot/gel image data are in Supporting Information or posted at a public data repository, provide the repository URL if relevant, and provide specific details as to which raw blot/gel images, if any, are not available.**

Reply：All the original data have been supplemented and uploaded to the supplementary file.

8. **Please upload a new copy of Figures 3 and 4 as the detail is not clear. Please follow the link for more information:**

Reply：I can assure you that the uploaded images are high-definition ones in accordance with the journal's requirements. However, after the PDF was generated, the images became very blurry. I have tried many methods but the situation remains the same. I'm very sorry. However, I have re-uploaded the pictures, hoping this will be helpful.

9. **If the reviewer comments include a recommendation to cite specific previously published works, please review and evaluate these publications to determine whether they are relevant and should be cited. There is no requirement to cite these works unless the editor has indicated otherwise.**

Reply：No similar problems occurred.

**Response to Reviewer 1：**

1. **Study design and experimental unit (critical)**

Reply：In the "2. MATERIALS AND METHODS" section, a new subsection "2.8 Experimental unit and replication design" has been added. The research design and experimental units were elaborately described. Line:203-210

2. **Sample size justification**

Reply：In the "2. MATERIALS AND METHODS" section, a new subsection "2.8 Experimental unit and replication design" has been added. It indicates that n is greater than or equal to 3, which means each experiment is replicated more than three times. Line:203-210

3. **Insufficient methodological detail (reproducibility issue)**

Reply：The primer sequences used in the study have been uploaded as supplementary files.

4. **Statistical analysis – incomplete and sometimes inappropriate**

Reply：The section on 2.7 Statistical Analysis has been revised again.

5. **Confounding factors not controlled**

Reply：In Section 2.1, the standardized control of experimental conditions is supplemented, and the aforementioned confounding factors are explicitly excluded: All cell cultures and virus experiments are conducted in a controlled laboratory environment. Line:196-202

6. **Overinterpretation of results**

Reply：Vague expressions such as “were associated with” and “showed higher” have been replaced with precise alternatives: “suggests”, “improved”, “resulted in” and “led to”.

7. **Introduction – needs a sharper knowledge gap Currently too descriptive.**

Reply：The introduction section was supplemented and revised as per the requirements, and a clear research objective was added at the end. Line:115-122

8. **Results section：repeats methods， includes interpretation**

Reply：Make the necessary revisions based on the opinions. Remove the repetitive descriptions of the methods in the results section and place all the explanatory content in the discussion section.

9. **Discussion – structure**

Reply：The discussion section has been revised in accordance with the comments, and the consistency of abbreviations, as well as the presentation and formatting of figures and tables, have been carefully reviewed and verified throughout the manuscript.

**Response to Reviewer 2：**

1. **Title**

Reply：It has been revised to: Identification of an Optimal Exogenous Gene Insertion Site (P–M) and Establishment of a Reverse Genetics System for aMPV/A	Line:1-2

2. **Abstract: The claim that vector vaccine development is hindered solely by the lack of a reverse geneticssystem and unclear insertion sites is inaccurate as it is not the only reason!**

Reply：It has been revised to: The development of highly efficient viral vector vaccines is constrained by multiple factors, among which the lack of a mature reverse genetics system and the uncertainty regarding the optimal insertion site for foreign genes represent one of the core technical bottlenecks. Line:21-24

3. **Abstract: Please include key quantitative results for example fold differences in EGFP expression, viral titer comparisons, and passage stability, etc.**

Reply：Based on the suggestions, revisions were made as follows: The results demonstrated that the expression level at the P‑M locus after EGFP insertion was approximately 1.2‑fold higher than that of other recombinant viruses. In addition, there was no significant difference in viral titer, and good genetic stability was observed over 20 generations. Line:27-33

4. **Also revise the sentence to: This study establishes a stable and efficient reverse genetics system for aMPV/A…**

Reply：Based on the suggestions, revisions were made as follows: This study establishes a stable and efficient reverse genetics system for aMPV/A, which provides a critical technical platform for aMPV/A molecular mechanism research and novel recombinant vector vaccine development, with important practical value for global aMPV prevention and control.

5. **Line 50-51 aMPV poses a significant threat to the global poultry industry, causing substantial50 economic losses[7]. can be moved to begingin of introduction**

Reply：Put in the first sentence of the first paragraph of the introduction. Line:39-40

6. **Line 48 revise as follow
   Subtypes A and B are the most widely distributed aMPV lineages globally, although their regional prevalence varies and other subtypes may predominate in specific geographic areas**

Reply：Based on the suggestions, revisions were made as follows: Subtypes A and B are the most widely distributed aMPV lineages globally, although their regional prevalence varies and other subtypes may predominate in specific geographic areas. Line:49-51

7. **The novelty is not clearly defined given prior insertion-site studies; What gap specifically exists for subtype A?**

Reply：Based on the suggestions, revisions were made as follows: Prior studies have identified optimal exogenous gene insertion sites and established reverse genetics systems for aMPV/C subtypes[17,18,28], whereas no systematic investigation has been conducted on insertion site selection for aMPV/A—a subtype that has recently re-emerged in North America (USA and Mexico) and caused large-scale outbreaks in the poultry industry[8,9,10]. To address this gap, the present study constructed a pBR322-T7 RNA polymerase dual-based aMPV/A reverse genetics system and systematically evaluated seven intergenic insertion sites, representing the first report on the optimal exogenous gene insertion locus for aMPV/A to date. Line:116-123

8. **2.1 Cell Line, Virus, Plasmids, and Nucleic Acid
   This subtitle does not makesense. Change it to "Cells" and explain the cell line after mentioning which viruses are used. Move the plasmid details to 2.2.**

Reply：According to the suggestions, the section "2.2 Construction of recombinant virus and auxiliary plasmids" has been supplemented with "Virus and nucleic acid information", "Genome design and compliance with the Rule of Six", "Details of gene start/stop and intergenic sequences", "Cloning strategy details" and "Supplement on plasmid construction". Line:136-159

9. **-Statistical analysis is also insufficiently described.**

Reply：Based on the revision suggestions, I have rewritten 2.7 Statistical Analysis and added 2.8 Experimental Unit and Replication Design. Line:197-211

10. **Results-Virus growth curve analysis revealed that…Do you mean Growth-curve analysis demonstrated that?**

Reply：The original sentence “Virus growth curve analysis revealed that…” was revised to: Growth-curve analysis demonstrated that. Line:214

11. **So not he question is whether the rule of six was maintained final genomelengths and whether EGFP insertions were adjusted accordingly?**

Reply：The 6-base principle has been added to the "Genome design and Rule of Six compliance" section in the "2.2 Construction of recombinant virus and auxiliary plasmids" subsection. Line:141-146

12. **The figures need more detailed captions**

Reply：Modify and supplement all the legends. Line:160-167;219-226;233-243;250-260

13. **English language editing is strongly recommended.**

Reply：Revise the entire text in English to make it smooth and fluent, following English expression conventions.
