# Supplementary material for: Identification of an optimal exogenous gene insertion site (P–M) and establishment of a reverse genetics system for aMPV/A
Source: PLoS One. 2026 Apr 20;21(4):e0347597. doi: 10.1371/journal.pone.0347597 (PMC13094954; doi:10.1371/journal.pone.0347597)
Supplement: Attachment — Submitted filename: R2.docx [file pone.0347597.s004.docx]

1. **Inconsistent virus nomenclature in Results section 3.1:** The text states *"rMPV/B-NP-EGFP"* and *"rMPV/B-GL-EGFP"* (page 46, lines 214-217), but the manuscript is focused on aMPV/A. This appears to be a typographical error; it should likely read *"raMPV/A-NP-EGFP"* and *"raMPV/A-GL-EGFP"* to be consistent with the rest of the document and Figure 2.
2. **Incorrect section numbering:** In the Results section, the subheadings for exogenous gene expression (page 46, line 233) and genetic stability (page 47, line 244) are numbered 2.2 and 2.3. These should be 3.2 and 3.3 to align with the main section numbering.
3. **Gene stop signal inconsistency:** In the methods (page 43, line 151), the gene stop signal is listed as 3'- tattagaaaaaa- 3', which appears to be a correction from the earlier version. However, the abstract and results still refer to the P-M locus. The authors should double-check that the correct gene start/stop sequences are consistently used throughout the manuscript and supplementary files.
4. **Figure 2 y-axis label:** The label currently reads *"logio TCIDso/mL"* (page 46, line 226). This should be corrected to *"log₁₀ TCID₅₀/mL"*.
