# Supplementary material for: Identification of an optimal exogenous gene insertion site (P–M) and establishment of a reverse genetics system for aMPV/A
Source: PLoS One. 2026 Apr 20;21(4):e0347597. doi: 10.1371/journal.pone.0347597 (PMC13094954; doi:10.1371/journal.pone.0347597)
Supplement: Attachment — Submitted filename: Response to Reviewers2.docx [file pone.0347597.s006.docx]

**Response to Reviewers**

**Response to Reviewer#1：**

**1. Inconsistent virus nomenclature in Results section 3.1: The text states "rMPV/B-NP-EGFP" and "rMPV/B-GL-EGFP" (page 46, lines 214-217), but the manuscript is focused on aMPV/A. This appears to be a typographical error; it should likely read "raMPV/A-NP-EGFP" and "raMPV/A-GL-EGFP" to be consistent with the rest of the document and Figure 2.**

Reply：Thank you for your feedback. The text has been updated to read "raMPV/A-NP-EGFP" and "raMPV/A-GL-EGFP" on lines 214-217.

**2. Incorrect section numbering: In the Results section, the subheadings for exogenous gene expression (page 46, line 233) and genetic stability (page 47, line 244) are numbered 2.2 and 2.3. These should be 3.2 and 3.3 to align with the main section numbering.**

Reply：Thank you for your feedback. The sequence numbers of the subheadings have been changed to 3.2 and 3.3. (This change has been made on lines 233 and 244.)

**3. Gene stop signal inconsistency: In the methods (page 43, line 151), the gene stop signal is listed as 3'- tattagaaaaaa- 3', which appears to be a correction from the earlier version. However, the abstract and results still refer to the P-M locus. The authors should double-check that the correct gene start/stopsequences are consistently used throughout the manuscript and supplementary files.**

Reply：Thank you for the correction. The "GS: 5'- ggacaagtcaaa-3' " and "gene stop (GS: 5'-ttatgaaaaaaa-3')" signals have been changed to lines 148-149.

**4. Figure 2 y-axis label: The label currently reads "logio TCIDso/mL" (page 46, line 226). This should be corrected to "****log₁₀ TCID₅₀/mL".**

Reply：Thank you for the correction. The y-axis of FIG2 has been modified to log₁₀ TCID₅₀/mL.
